# Reputation based on internal capabilities: The case of small enterprises within the Colombian orange economy

**DOI:** 10.1371/journal.pone.0285026

**Published:** 2023-06-21

**Authors:** Yonni Angel Cuero-Acosta, Marelby Amado-Mateus, Daniel Ricardo Torralba Barreto, Suelen Emilia Castiblanco-Moreno

**Affiliations:** 1 Business School, Universidad del Rosario, Bogotá, Colombia; 2 UR STEAM, Universidad del Rosario, Bogotá, Colombia; 3 Centro de Estudios para la Competitividad Regional, Universidad del Rosario, Bogotá, Colombia; 4 School of Economics and Administrative Sciences, Corporación Universitaria de Asturias, Bogotá, Colombia; Instituto Politecnico Nacional, MEXICO

## Abstract

This study seeks to understand how micro, small, and medium-sized enterprises (MSMEs) can be innovative while developing their reputation. In doing so, this study focuses on companies classified as promoters of Colombia’s “orange economy,” which relates to the country’s cultural and creative diversity. Firms with a non-technological emphasis also require knowledge, innovation, and reputation to perform better. In this regard, the study is based on the work of Hormiga and Garcia-Almeida (2016) who proposed the relationship between accumulated knowledge and innovation as background of reputation. In this paper, the purpose is to understand the internal reputation-building process carried out by MSMEs and the variables that intervene. Therefore, this research accounts for how MSMEs can build their reputation through innovation and accumulated knowledge. For this, a survey was conducted in 320 orange economy MSMEs located in Bogotá, Colombia, and the relationship of these variables was statistically tested through a quantitative analysis of multivariate data. Innovation was found to not impact the performance of companies, but this can be associated to factors that were not considered in this research. However, it is proposed to refine the original model by considering the manager’s perspective. It is recommended that entrepreneurs invest resources in accumulating internal (tacit) knowledge to improve skills that enhance reputation.

## Introduction

Micro, small, and medium-sized enterprises (MSMEs), are growing considerably in Colombia; according to the Confederación Colombiana de Cámaras de Comercio (Confecámaras) [1], 309,463 enterprises were created in Colombia in 2019, which represents a 2.1% increase from 2018 (303,027). According to the Confecámaras, companies belonging to the orange economy grew even more: “it was found that during 2019 a total of 9,122 companies were created, that is, a growth of 7.7% in this segment compared to the same period last year” [1]. Companies in the orange economy have gained importance in Colombia as a result of their successful exploitation of the talent and cultural wealth of the country to drive economic growth. Hence, different mechanisms have been implemented to favor their development of the sector. However, companies in the orange economy sector are not immune to the challenges typically faced by newly created companies, including being acknowledged by others in the market and ensuring better business performance.

An additional phenomenon has happened in Colombia in the last few years. The 2018 closure and bankruptcy figures are striking, causing the favorable business situation to be reconsidered since approximately 180,507 enterprises in Colombia went bankrupt [2]. In general, these statistics reveal that about 50% of the companies created that year had to close down. Although Colombian companies have a strong entrepreneurial and productive capacity, it is reasonable for nascent companies to be concerned with their economic sustainability. In addition, companies need to develop and implement strategies that allow them to reach their target market. In other words, there are various challenges for nascent companies, and the salient ones in an emerging economy include financial leverage, logistics, hiring specialized personnel, and gaining recognition in the sector in which they operate.

Regarding recognition, MSMEs must be cautious with their use of resources to build their reputation. As a rule, businesses invest in their image to build a reputation; however, this does not positively impact their performance. Empirically, in the Colombian context, when small companies are seen to be doing things differently, they capture more customers in a defined market. Therefore, companies must communicate the innovations developed to the market before they can see a better result in their operations. Previous studies established that companies can turn their internal capabilities into assets to build their local reputation in the market [3].

Theoretically, the resource-based view (RBV) framework explains the role of skills within the companies. For instance, knowledge is fundamental to being considered the heart of business management [3,4]. To date, the RBV theory reveals that companies manage their capacities to develop higher-order skills, which gives them a competitive advantage [5]; this is especially true in companies that are not knowledge intensive. While large companies could invest directly in building their reputation in a particular market, small companies need to concentrate on developing skills that will help them build a local reputation. More evidence on how this process occurs, what it implies for organizational performance, and how small businesses can benefit from the interaction between their internal capabilities and the establishment of a reputation is required.

Therefore, we assume that, in the case of MSMEs, the focus on internal capabilities is the first step in determining the company’s success in terms of reputation. We seek to understand how these capabilities, such as accumulated knowledge and innovation capacity, are related to reputation building and organizational performance in MSMEs, which are not technology intensive. This research contributes to the resolution of the following question. What is the relationship between knowledge, innovation, and reputation within MSMEs in the orange economy?

## Theoretical framework

### 2.1 MSMEs’ reputation

According to the existing literature, reputation is related to large companies and how they communicate their fulfillment of the promised value and corporate principles to each stakeholder. The term corporate reputation was thus coined, referring to stakeholder acknowledgment of organizational behavior and performance [6,7]. Thus, being acknowledged is a priority and challenge, especially for new companies that must do it as soon as possible. Even though new firms need to build a reputation quickly, reputation in itself is not built in the short term, as it results from the combination of long-term investments, such as money, market image, increase in sales, or profitability [8]. Some authors, highlight that corporate reputation has its theoretical roots in marketing and is based on contributions from stakeholder theory and other fields, such as entrepreneurship, advertising, and organizational behavior theories [9]. Reputation includes both corporate image and identity, the former from external stakeholders and latter from internal stakeholders [10]. Thus, it is complex and involves a process wherein reputation is built and assessed from the inside out [9]. Therefore, theorists define corporate reputation as a branch of marketing in which the way a company relates to and communicates with its environment influences how stakeholders perceive it [11].

When reputation is analyzed as a result of several forms of investment [12,13], it becomes clear why this concept is much more prevalent in large companies that possess the resources and personnel to turn reputation into a company’s strategic asset. This does not imply that small companies do not build a reputation. On the contrary, the current boom of social media allowed MSMEs to generate an immediate and direct connection with their stakeholders [14], communicating not only their product attributes but also the methods of production considering global conservation trends in their environment. Following this line of thought, particular studies determined that “a higher number of social network channels used for customer service is associated with a higher reputation score for a firm” [15]. Social media have helped mitigate the disadvantages of financial muscle that small enterprises face compared to large enterprises [16].

On the contrary, reputation has provided large companies with additional benefits, such as the vehicle to legitimize a company’s brand [17,18]. Consequently, corporations spare no marketing efforts for brand positioning. Even if they suffer any inconvenience in production or distribution processes, marketing mitigates these negative factors and advocates for and defends the brand [19].

Reputation for MSMEs is a driving factor in attaining profitability [20]. In emerging countries, the capacity to generate strategic thinking is limited, as the context exerts pressure to achieve quick results [21]. Thus, MSME entrepreneurs build a reputation based on investment in image and decide on specific actions such as word-of-mouth (WOM) advertising to position their brand [8,22]. Nevertheless, the limited number of employees or financial resources make it necessary for MSMEs to enhance any resource usage. For these companies, how they do so is relevant if they are to boost the impact of any investment. For instance, a priority in a small company is to develop its operational capabilities to ensure its performance. While this is true, existing studies have not paid much attention to how reputation may result from the company’s operational capabilities. Clearly, the acknowledgment and independent effort in which the direct investment is made will eventually enhance reputation indirectly but safely and consistently. This is an alternative and logical method to be followed by MSMEs rather than investing in image and communication. Hence, if a company has limited resources, its priorities must focus on consolidating its know-how, that is, the fundamental basis of its competitive advantage. Priorities will thus focus on building and consolidating organizational knowledge to innovate and subsequently be recognized for this capability.

### 2.2 Knowledge and innovation within MSMEs

Knowledge is considered a company’s primary strategic resource [23], which generates a sustainable competitive advantage [24]. Over the last few decades, due to the massive access to information, knowledge has become an essential commodity for countries’ economic growth as well as for companies’ competitive advantages at the organizational level [25]. Thus, knowledge and its management are part of the inputs required to innovate. The entrepreneur is the first source of knowledge for companies [26]. Several factors influence an entrepreneur, expert, or businessperson’s knowledge, and it is not just governed by the formally gained knowledge [27]. Previous research shows that innovation in new ventures is directly influenced by the characteristics of business personnel, including their education and entrepreneurial experience [28,29] both as an entrepreneur and an employee [30]. This provides better knowledge of the sector (they are an expert in the area), market, or access to business networks [31]. However, being experienced also brings disadvantages for personnel, as knowledge may influence their decisions and hinder the innovative process, turning them into imitators [29]. Despite this risk, companies recognize that the entrepreneur is a source of knowledge. Entrepreneurs’ vision helps articulate their knowledge with the associates’ knowledge and market information [26].

Knowledge is a combination of experience, contextual information, and expert insight that provides a framework for evaluating information and the quality of experiences [32]. In this regard, knowledge may result from experiential learning within an organization, which is mediated by its culture, standards, and structure [4]. Another approach identifies knowledge as tacit and explicit and mentions that exchanging these two types of knowledge facilitates organizational innovation and performance [33]. A study conducted by Ganguly et al. [34] showed that the exchange of tacit and explicit knowledge and an organization’s innovative capability might stand out from the competition through its reputation for innovation. Thus, there is a continual dialogue between implicit and explicit knowledge, achieving a knowledge absorption spiral and generating the company’s absorptive capability, which subsequently becomes the basis for the process, area, product, or market innovation.

Furthermore, heterogeneous experiences enable knowledge sharing to have a positive impact on business performance and innovation. A study by Cheng et al. [35] showed that innovation is related to and contingent on knowledge acquisition and sharing capabilities. Within a firm, individuals and their interactions make innovations possible.

In particular, tacit knowledge is intuitive and acquired mainly through experience [36]. Although it is not formalized, its importance as an asset within the organization is recognized, depending on the interaction sector involved. According to du Plessis [37], tacit knowledge and knowledge management, in general, play a fundamental role in innovation. Other studies have focused on knowledge management and improving organizational innovation capability [38,39]. Similarly, Fletcher and Harris [40] discussed that internal and objective knowledge creates synergies within the organization, representing the competitive advantage of companies that know how to do something differently so that other firms cannot imitate. Therefore, knowledge and innovation are both required in any productive sector.

To understand the growth of companies in emerging countries, for example Colombia [41], the study of knowledge and innovation is a top priority. Global Entrepreneurship Monitor (GEM) Colombia 2019 [42], shows that there are different types of businesspersons: for example, in stage 2 (In the case of Colombia, the GEM methodology comprises stages 1, 2, 3, and 4) there are potential businesspersons who witness market opportunities where there is unfulfilled demand and set up their companies based on their knowledge, skills, and previous experience to fit such a demand. That study is focused on understanding the role of knowledge following the 2019 approach in which a GEM study included four new variables (vision, proactivity, opportunism, and innovation) to measure businesspersons’ skills. Innovation stood out as respondents stated they have excellent skills for innovation, with an 84.4% acceptance. However, the same survey showed that the measurement of social and cultural standards in Colombia is immaterial in promoting innovation. Therefore, to a greater extent, innovation in Colombia is based on companies and their members’ skills rather than on a promotion mechanism.

Although the skills of individuals are the core of new firms, this has not affected the products’ innovation. Established companies and new firms have a meager percentage of products deemed a novelty by their closest customers, ranging from 15.90% to 23.40% [42]. This means that for Colombia to have a more sophisticated production, it will be necessary to promote businesspersons’ capability to add more value to national production through innovation. The lack of innovation has led to a large number of nontechnology-based ventures in Colombia. During the last presidential term (2018–2022), Colombia’s development policy promoted the orange economy, thus encouraging creative and recurring capability through the legalization and incorporation of industrial companies, such as audiovisual companies, museums, and arts management. This focus on creativity is not a response to the inability to create technology but rather an attempt to harness the capability to innovate based on knowledge and experience, using an approach that utilizes individuals’ skills and talent and does not require a high level of complexity.

### 2.3 Innovation in new ventures and reputation

MSMEs and even startups apply marketing strategies to combine the development and implementation of innovation processes [43]. Innovation is key to organizational performance, and the outcome is evidenced in markets by introducing new products or ways of doing things [44]. New ideas are developed more quickly in established companies as they have greater access to financing than startups [45].

Innovation consolidates the establishment of companies and causes organizations to strengthen their reputation. This type of company receives market legitimacy for its creativity and ability to be at the forefront of product and process management [46]. In addition, an innovative company’s reputation becomes an intangible asset and a promise of sustainable competitive advantage [47]; although companies do not focus on being innovative, they gain recognition in a specific industry [48–50]. Companies grow and capitalize on commercial opportunities [51] as a result of their innovation capability, which consolidates them in a given market [46,52,53].

Previous studies contribute to examining the relationship between innovation and reputation. According to Harun et al. [54], this relationship boosts organizational performance and competitive advantage in the banking sector. Furthermore, Manohar et al. [55] infers a strong relationship between innovation and reputation in the service sector, which benefits from WOM advertising. Other studies find applications of the relationship between innovation and reputation in identifying the effect on purchase intention in the telecommunication sector [56]. Fombrun et al. [57] made a great theoretical contribution by generating innovation-based models to predict reputation. A company will achieve market success not only when it is innovative but also when it builds a reputation based on such capability or knowledge [58]. Thus, knowledge and innovation contribute to technological progress [59].

In this regard, the academic literature reports the capability of innovation to drive company reputation and customer loyalty [60]. Investing in innovation broadens the company’s capabilities [34]. According to Foroudi et al. [61], a company’s capability to attain a strong market position depends on other attributes, such as customer experience and how much experience may or may not be measured by technology. Thus, a company’s reputation as innovative must be thought of strategically. In this regard, Fuertes-Callén and Cuellar [62] found that the marketing and reputation of an innovative product are part of the relationship between product innovation and market success. Innovation enhances corporate reputation, and satisfied customers generate positive WOM [55]. Notwithstanding its importance, this relationship requires more profound analysis, especially for companies with limited financial capabilities. Thus, under the model proposed by Hormiga and García-Almeida [3], the following hypothesis is put forward:

H1. innovation is positively related to reputation.

### 2.4 Reputation and knowledge

Reputation communicates organizational capabilities, knowledge, reliability, and performance to the market, attracting more valuable customers and partners [63,64]. It also drives companies to search for innovation constantly, for which necessary updates and search for new knowledge are required [65,66]. Reputation, accumulated knowledge, and an organization’s specific needs allow for discovering and generating opportunities to strengthen business relationships and build networks [67]. Both reputation and knowledge are intangible assets that contribute to building a sustainable competitive advantage; however, they require time to develop correctly [68].

Knowledge also arises from the exchange between companies and people within the same company. According to Ganguly et al. [34], understanding marketing and innovation capabilities in developing a reputation for innovation is fundamental. However, knowledge sharing also plays a major role that may help build strategies consistent with a company’s characteristics. In this regard, Wang et al. [69] states that knowledge sharing among companies has a material impact on reputation and helps maintain a competitive advantage. Entrepreneurs’ accumulated knowledge has also been studied, although not so broadly. It was found that knowledge is influenced by past experiences, especially positive ones discarding the ones that failed [70], and entrepreneur knowledge is the key to building a positive reputation, especially for new ventures [3].

Research conducted by Brown et al. [71] showed a conceptual framework related to tangible and intangible assets and marketing capabilities. Within this framework, there is a relationship between intellectual and emotional assets (that comprise intangible assets), which include perceived quality, knowledge and competence, corporate reputation, and trust. For these authors, companies are differentiated by their capability or ability to exploit these (tangible and intangible) resources through different combinations and synergistic ways. In the case of creative companies, entrepreneurs consider it necessary to create an organization to realize their cultural and creative aspirations and generate a positive reputation by acknowledging their creativity [72].

As reviewed in connection with the relationship between entrepreneur knowledge and reputation and following the model proposed by Hormiga and García-Almeida [3], we developed the following hypothesis:

H2: Entrepreneur knowledge has a positive impact on reputation.

### 2.5 Reputation and performance

A company’s success is measured by its performance, that is, the outcome of the business relationship it builds to deliver products and services. It also depends on product quality and innovation, which must be unique, add value, and compete in the market. Thus, performance in a specific sector or market results from a company’s capability to position its profile as an innovative company through reputation [73]. According to Delgado-Verde et al. [74], corporate reputation significantly affects the interaction and complementarity between internal and external innovation activities. Thus, the better a company’s reputation, the more positive the interactive effect of internal research efforts, the development of new knowledge and external knowledge gained from product innovation.

From the stakeholders’ perspective, reputation is also an asset built socially, created and maintained through a legitimation process that entails a continuous and thorough assessment of the organization’s performance in each period, garnering its reputation [75,76]. Similarly, reputation is essential to a company’s performance and success [77]. In addition, certain studies have found that an experienced CEO, that is, one with business knowledge and experience, can maximize organizational reputation and efficiency or performance [78].

Recent and ongoing research shows that corporate reputation is probably the most important intangible asset that directly impacts organizational performance [68,79]. Research reported that innovative performance is substantially related to the reputation for technological innovation [80]. According to Low and Robins [64], reputation can materialize business performance or efficiency in the industrial environment and financial markets. Similarly, corporate reputation can be the key to achieving greater organizational resilience and performance [81]. As a result, pursuing a model proposed by Hormiga and García-Almeida [3], we formulated the following hypothesis:

H3: There is a positive relationship between reputation and corporate performance.

## Methodology

### 3.1 Design

This study seeks to understand the underlying processes that occur in the strategic management of companies. In this sense, authors such as da Costa Juniór et al. [82] have stated that the RBV theory arises in strategy and stands out mainly in management studies. Consequently, it is usual to reference the RBV theory to analyze an organization’s management behavior regarding tangible and intangible assets to define strategies that affect results [83,84]. To meet the objective of this study, it is necessary to understand the occurrence of the relationships raised through hypotheses from the reality of how they occur considering a truth-seeking research approach, which requires addressing quantitative methods [85].

This research design is based upon the theoretical foundations of concepts such as entrepreneurs’ knowledge and the conception of innovation, seen from applying strategic management processes. These require information to be provided by managers. Unfortunately, the effects of the COVID-19 pandemic measures in force in the companies at the time did not allow the entry of non-payroll personnel. Therefore, a virtual survey was conducted. This was accompanied, or in some cases, replaced, by telephone calls. This strategy allowed researchers to obtain first-hand relevant information regarding the conceptual assumptions, which guaranteed that answers were related to the meaning of the investigated construct.

To evaluate the consistency of the adapted scale to measure the four latent variables, techniques of exploratory and confirmatory analysis were selected based on Hormiga and García-Almeida [3], who pointed out that these types of analysis allow identifying the internal structure of a test and validating its capacity to measure what is expected adequately.

Structural equation modelling (SEM) is a technique that allows performing multivariate confirmatory analyses rather than just descriptive ones [86]. Moreover, as stated by Novikova et al. [87, p. 146–147], “SEM has three major advantages over traditional multivariate techniques: (1) explicit assessment of measurement error; (2) estimation of latent (unobserved) variables via observed variables; and (3) model testing where a structure can be imposed and assessed as to fit of the data.”

For this study, following the theoretical framework, variables of interest are latent variables that need a subset of observed variables to be approached, and SEM modeling permits a more precise assessment of these variables. Thus, SEM modeling has been widely used in the empirical literature on reputation and innovation.

## 3.2 Materials and methods

### 3.2.1 Sample

In this research, the sample included companies formally incorporated in the Chamber of Commerce of Bogotá (CCB), Colombia, over the past five years, 2016–2021 [88]. The CCB states in the business dynamics report that Colombia’s capital city “leads the establishment of companies and has the highest percentage of high-impact ventures in Colombia” [88]. The type of companies selected is MSMEs, units of economic exploitation by an individual or legal entity in business, agricultural, industrial, commercial, or service activities, whether rural or urban. Each enterprise has specific parameters ranging from total headcount to total assets to identify the category to which they belong (Law No. 590 of 2000).

Of the total number of companies established, we chose entities operating within the orange economy, due to their importance as economic growth drivers. In the second half of 2019, companies engaging in the provision of cultural and creative services were becoming the cornerstone of Colombia’s social and economic transformation, which amounted to 1,956 companies. All International Standard Industrial Classification of All Economic Activities (ISIC) codes were considered in this sample, including news agencies and other information systems, audiovisual and publishing activities, photography, digital media, software, and advertising.

The orange economy became relevant in Colombia after the Creative Economy Promotion Law (or Orange Economy Law) was enacted on May 23, 2017, aimed at “developing, promoting, encouraging, and protecting creative industries” based on intellectual property to create value [89]. The Creative Economy Report issued by the United Nations Conference on Trade and Development identifies three cultural industries: artistic, scientific, and economic creativity. According to the Colombian Ministry of Culture [90], “…orange economy activities comprise the arts and tangible and intangible cultural heritage, cultural industries, and functional creations” (p. 8). Artistic and heritage activities include visual and performing arts; tourism and cultural heritage; education; cultural industries, including publishing companies, photography, and audiovisual activities; and functional creations, new media, and content creation software, including digital media, design, and advertising.

The sample was selected by simple random sampling with a 5% margin of error and 99% confidence interval. Therefore, the instrument was applied to a sample of 500 companies (three more than calculated) randomly selected from the complete list of 1,956 companies in the sector. In total, 1,956 questionnaires were sent to the companies, and 27% of them were answered. Among them, 500 questionnaires were useable for the analysis. To characterize the sample, 21.20% of the surveyed companies belonged to the commerce economic sector, 54.60% had between 11 and 50 employees, 35.40% were three years old, and 39.80% of surveys were answered by companies’ managers.

#### Questionnaire

This research used a structured questionnaire developed by Hormiga and García-Almeida [3] to evaluate five variables: 1) concern for new technology, 2) product/service innovation, 3) the entrepreneur’s accumulated knowledge, 4) reputation, and 5) new venture performance. In total, the instrument has 20 items, with the 1) dimension having three items, the 5) has five items, and the other three 2), 3), and 4) four items each. It uses a 5-point Likert-type scale with 1 = strongly disagree, 2 = disagree, 3 = neither disagree nor agree, 4 = agree, and 5 = strongly agree. As mentioned in the following section, data analysis, the instrument was adapted to the Colombian context.

#### Data collection

In light of the global pandemic, the instrument was applied virtually. In addition, telephone calls were made to creative companies connected with the orange economy. The surveys were administered in the period between July and August 2020.

As pointed out earlier, the questionnaire used the same scale to measure the dependent and independent variables; this situation, called the common method bias (CMB), may threaten the results’ validity and reliability. Following Kock et al. [84], CMB was controlled using procedural and statistical controls.

Regarding procedural controls, several steps were followed; participants in the survey received clear instructions, the anonymity of responses was ensured, complex and ambiguous items were avoided, and the survey was short and straightforward. Moreover, psychological separation was implemented, meaning the causal link between the independent and dependent variables was masked.

On the other hand, Hartman’s single-factor test was carried out. This test points out that there are CMB problems if the unrotated solution produces one factor that explains more than 50% of the variance. In fact, the results of the unrotated solution showed that the factor with the highest contribution explains the 27% of the factors variance.

#### Data analysis

The instrument used in this study was designed for a population of non-high-tech industries from the Canary Islands (Spain). Consequently, it was necessary to develop a cultural adaptation process. In accordance with Beaton et al. [85], two steps were followed. First, the original instrument was translated and adapted to the Spanish language; second, the instrument was reviewed by a committee of experts consisting of five marketing and entrepreneurship professionals who reviewed the questionnaire and evaluated its pertinence, relevance, and clarity of wording. These aspects were evaluated on a scale of 1–5, where 1 is the lowest and 5 is the highest. The experts’ judgments were analyzed through Aiken’s V statistic (V) [91], which uses a scale between 0 and 1, where 0 represents a generalized disagreement and 1 a perfect level of agreement. The results showed that pertinence was V = 0.82, relevance V = 0.87, and clarity of wording V = 0.92, showing a good level of agreement among the judges.

After guaranteeing the content validity of the instrument, statistical tests were performed. Following Hormiga and García-Almeida [3], a factorial exploratory analysis was used to evaluate the structure of the latent variables of interest in this study and their behavior in the Colombian case. This analysis allowed us to identify how many original items should be preserved and how strong their relationship with the latent variables was.

The exploratory factor analysis (EFA) was performed using principal component analysis (PCA) [92] and a Varimax rotation since it is assumed that the dimensions are unrelated [3]. The sample collected in this research was split into two groups, so the EFA and confirmatory factor analysis (CFA) were performed with two different samples [93].

The obtained structure was tested through CFA. Since data is not normally distributed, the estimation of CFA was carried out through the maximum likelihood method using robust methods [94].

Finally, a structural equation model was developed to estimate the influence of innovation, reputation, and entrepreneur knowledge over companies’ performance in Colombia’s creative industry. SEM tests and evaluates multivariate causal relationships [95]. It has become one of the most powerful tools for evaluating relationships between social phenomena since it is based on the psychometric tradition of using observed variables to measure latent and theoretically complex variables. Considering this, structural equation models simultaneously test several interrelated dependency relationships and predict multiple dependent variables [96].

The statistical analysis was developed using the software R Studio v. 1.1.456, and the packages psych v. 1.8.12 [97], lavaan v.0.6–11 [98], and semTools [99]. The code and data can be found in Annex 1. The results of these analyses are presented in detail in the Results section.

## Results

### 4.1 Analysis of the factors

The original instrument [3] analyzes five dimensions: technology, new venture performance, reputation, innovation, and the entrepreneur’s accumulated knowledge. Even though these variables and their measurements were validated for 130 Spanish companies, there are significant differences between the Spanish and Colombian contexts and the companies included in the sample of the original instrument validation. Therefore, it was necessary to conduct a new analysis that helped to identify whether the original items and latent variables were appropriate for the Colombian case. First, EFA was used; then, the preserved items were analyzed through CFA to assess construct validity. The EFA was performed using PCA [92] and a Varimax rotation since it is assumed that the dimensions are unrelated [3].

Following the recommendations of Field [92], the sample collected in this research was divided into two groups, so the EFA and the CFA were performed with two different samples. The original instrument was built upon 20 items: three for technology, four for innovation, four for entrepreneur’s knowledge, four for reputation, and five for performance. First, we estimated the Kaiser Meyer Ohlin (KMO) to test the adequacy of the sample to perform the EFA [92]. Results suggested eliminating the three items of the technology dimension and two items of the knowledge dimension because KMO values were below 0.7; therefore, we kept 15 items with a KMO of 0.82, which is evidence of very good adequacy [100].

In addition to the KMO, we also tested the hypothesis that the correlation matrix was an identity matrix; in other words, variables included in the analysis do not correlate. Bartlett’s test evaluates the null hypothesis that the correlation matrix is an identity matrix; thus, as our results suggested rejecting the null hypothesis [890.1703(105)***], we can conclude that correlation between variables are different from zero [92]. However, the opposite, a very strong relationship between variables, can also be problematic because it can be evidence of extreme multicollinearity. To test the existence of this singularity, we used the determinant of the R-matrix, which we expected to show a result greater than 0.00001, to verify that variables are not highly correlated [92]. For our case, this determinant was 0.0257.

After these preliminary steps, an initial analysis was run to obtain each factor’s eigenvalue. The scree plot suggested preserving four dimensions, which explain 57% of the variance. Table 1 shows the factor loadings after the *Varimax* rotation. The items that cluster in the same factor suggest that dimension 1 represents “Performance,” dimension 2 represents “Innovation,” dimension 3 represents “Reputation1,” and dimension 4 represents “Reputation2.”

**Table 1:** Synthesis of exploratory factor analysis (n = 250).

	Item	Factor loading			
		Varimax Rotation			
		PER RC1	INN RC2	REP2 RC4	REP1 RC3
PER03	What is your degree of satisfaction regarding the attainment of the original objectives of your company?	0.77			
PER02	What is your degree of satisfaction regarding the return on investment of your company?	0.73			
PER04	What is your degree of satisfaction regarding the overall success of your company?	0.73			
PER01	What is your degree of satisfaction regarding the volume of sales of your company?	0.70			
PER05	What is your degree of satisfaction regarding the success of your company compared with competitors?	0.65			
INN03	Has the company improved its products and services in response to the needs of clients?		0.78		
INN01	Can the company adapt easily to changes in the business environment?		0.77		
INN02	Has the company improved its products and services in response to the suggestions of its clients?		0.71		
INN04	Do the comments and recommendations of our clients usually lead to changes in the organization?		0.64		
REP02	Compared to my competitors, has the reputation of my company increased significantly?			0.72	
KNW03	Number of years of experience in this sector (as an entrepreneur and/or employee)			0.70	
REP01	Has the company attained an excellent reputation during the early months/years of its existence?			0.65	
REP04	What proportion of clients are repeat customers after the first purchase or service?				0.80
REP03	What proportion of clients do you think will recommend your company?				0.62
KNW02	Much of the knowledge and skills that I require for my present business are the same as I required for my previous work activity				-0.48
Eigenvalues		2.93	2.43	1.67	1.46
% of explained variance		0.20	0.16	0.11	0.10
Cronbach’s alpha		0.79	0.73	0.34	0.55

Extraction method: Principal component analysis; rotation method: Varimax normalization with Kaiser; fit based upon off-diagonal values 0.9. Loadings below 0.30 are not shown.

Cronbach’s α was computed for the general instrument (0.79) and for each one of the four dimensions: Performance (0.79), Innovation (0.73), Reputation1 (0.34), and Reputation2 (0.55).

The results showed a different structure than that proposed by Hormiga & García-Almeida. Although the innovation (INN) and new venture performance (PER) dimensions in the factorial analysis had similar Cronbach’s alpha values to the original ones, the other dimensions changed. The reputation dimension was split into two dimensions, one that captures the subjective views of the surveyed companies about their reputation (REP1) and another that captures more objective dimensions of reputation (REP2). It is noteworthy that both reputation dimensions include one item initially intended to capture the entrepreneur’s knowledge. In other words, those knowledge items are not statistically different from the ones that evaluate reputation; therefore, the existence of the entrepreneur’s knowledge dimension was not confirmed.

After obtaining an initial factor structure, Table 2 shows the descriptive statistics for each dimension extracted from the analysis. Results are similar to those reported by Hormiga and García-Almeida [3] and are evidence of high score variability.

**Table 2:** Descriptive statistics.

	*n*	Minimum	Maximum	Mean	SD
Innovation	500	10	21	22,64	2,80
Reputation 1	500	5	21	16,37	2,88
Reputation 2	500	3	21	16,20	2,23
Performance	500	5	35	27,31	4,01

This initial structure was tested through CFA. Since data is not normally distributed, the estimation of CFA was carried out through the maximum likelihood method using robust methods [101]. Fit model measurements show a good model according to the absolute criteria of Standardized Root Mean Square Residual (SRMR = 0.047 <0.08) [102] and the Comparative Fit Index criteria (CFI = 0.981> 0.90). The model also indicates a good fit in the parsimony test Root Mean Square Error of Approximation RMSEA = 0.029<0.05) [103].

Afterward, the validity of the instrument was assessed. Convergent and discriminant validity were evaluated (Table 3). For convergent validity, it was evaluated that the average of the standardized loads on a factor was around 0.7 and that the individual factor loads were higher than 0.6 [94]. This criterion was partially met because the factorial loads of items INN04, KNW03, and REP04 are slightly lower than 0.6. However, the items were preserved due to theoretical reasons.

**Table 3:** Convergent validity, composite reliability, and average variance extracted.

Factor	Indicator	Standardized loadings	CR	AVE
INN	INN01	0,732***	0,7	0,4
	INN02	0,568***		
	INN03	0,730***		
	INN04	0,495***		
REP2	REP01	0,723***	0,6	0,4
	REP02	0,668***		
	KNW03	0,309***		
REP1	REP03	0,785***	0,6	0,4
	REP04	0,484***		
PER	PER01	0,590***	0,7	0,4
	PER03	0,644***		
	PER04	0,721***		
	PER05	0,659***		

Note: Chi2 (59) = 71.231; CFI = 0.981; TLI = 0.975; RMSEA (90%CI) = 0.029 (0.000; 0.051); CR = Composite Reliability; AVE = Average Variance Extracted.

In addition to standardized load assessment, convergent validity was tested using the Average Variance Extracted (AVE). This indicator shows results below the recommended standard (0.5); however, the second criterion to evaluate the convergent validity (composite reliability) shows good results (>0.6) [94].

After convergent validation, discriminant validity analysis was carried out using the confidence interval test [93]. This test verifies that the value “1” is not contained in the confidence interval of the correlations between the different dimensions.

As Table 4 indicates, the results of the measurement of discriminant validity through the confidence interval test are evidence of the existence of divergent validity.

**Table 4:** Divergent validity—confidence interval test.

Correlations	Standardized loadings	Error	Confidence Interval -2	Confidence Interval +2
INN~~REP1	0,367	0,082	0,531	0,203
INN~~REP2	0,251	0,089	0,429	0,073
INN~~PER	0,345	0,077	0,499	0,191
PER~~REP1	0,620	0,069	0,758	0,482
PER~~REP2	0,450	0,086	0,622	0,278
REP1~~REP2	0,555	0,091	0,737	0,373

### 4.2 Structural equation model

**Fig 1:**
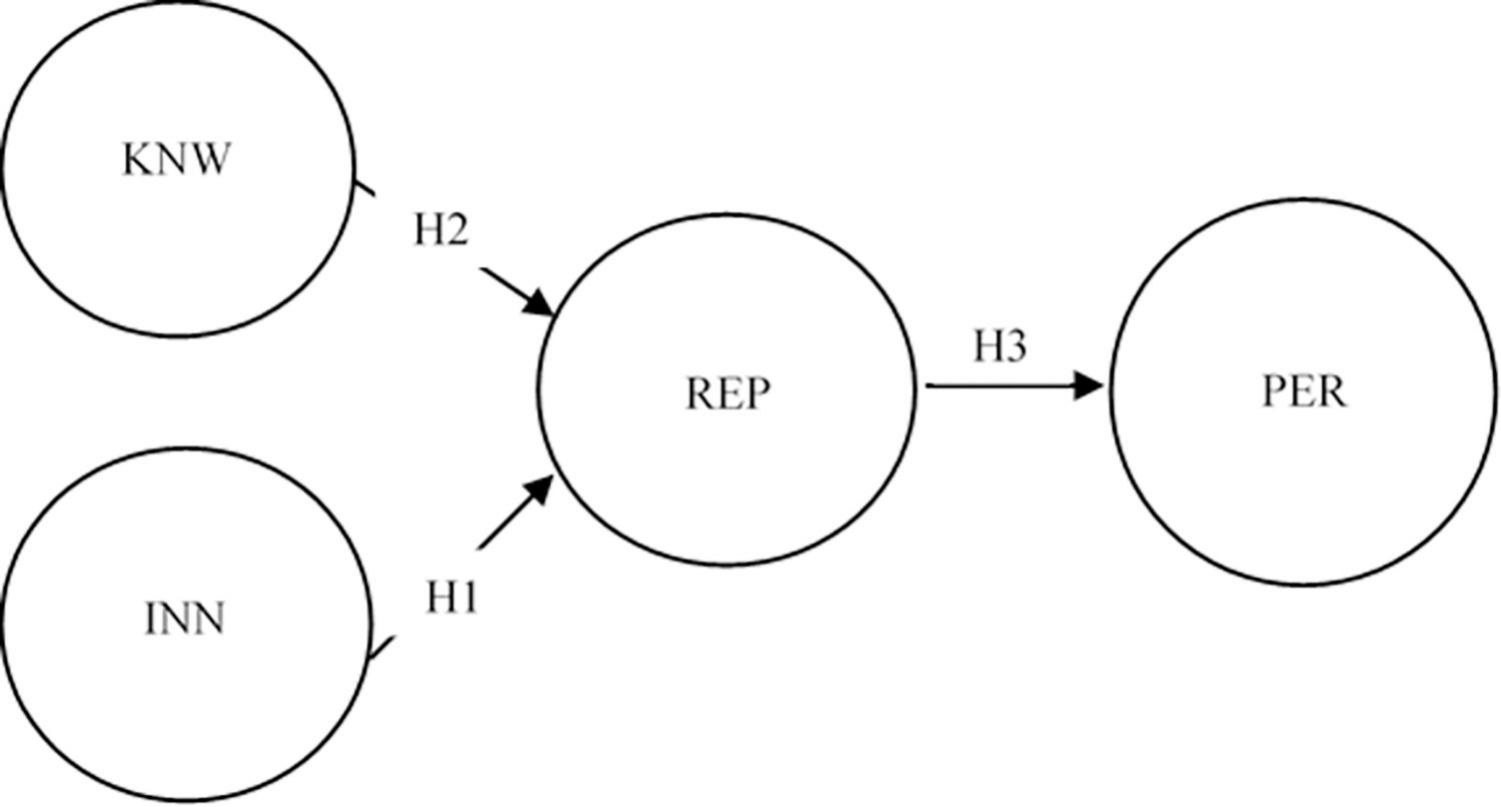
Synthesis proposed theoretical model.

**Fig 2:**
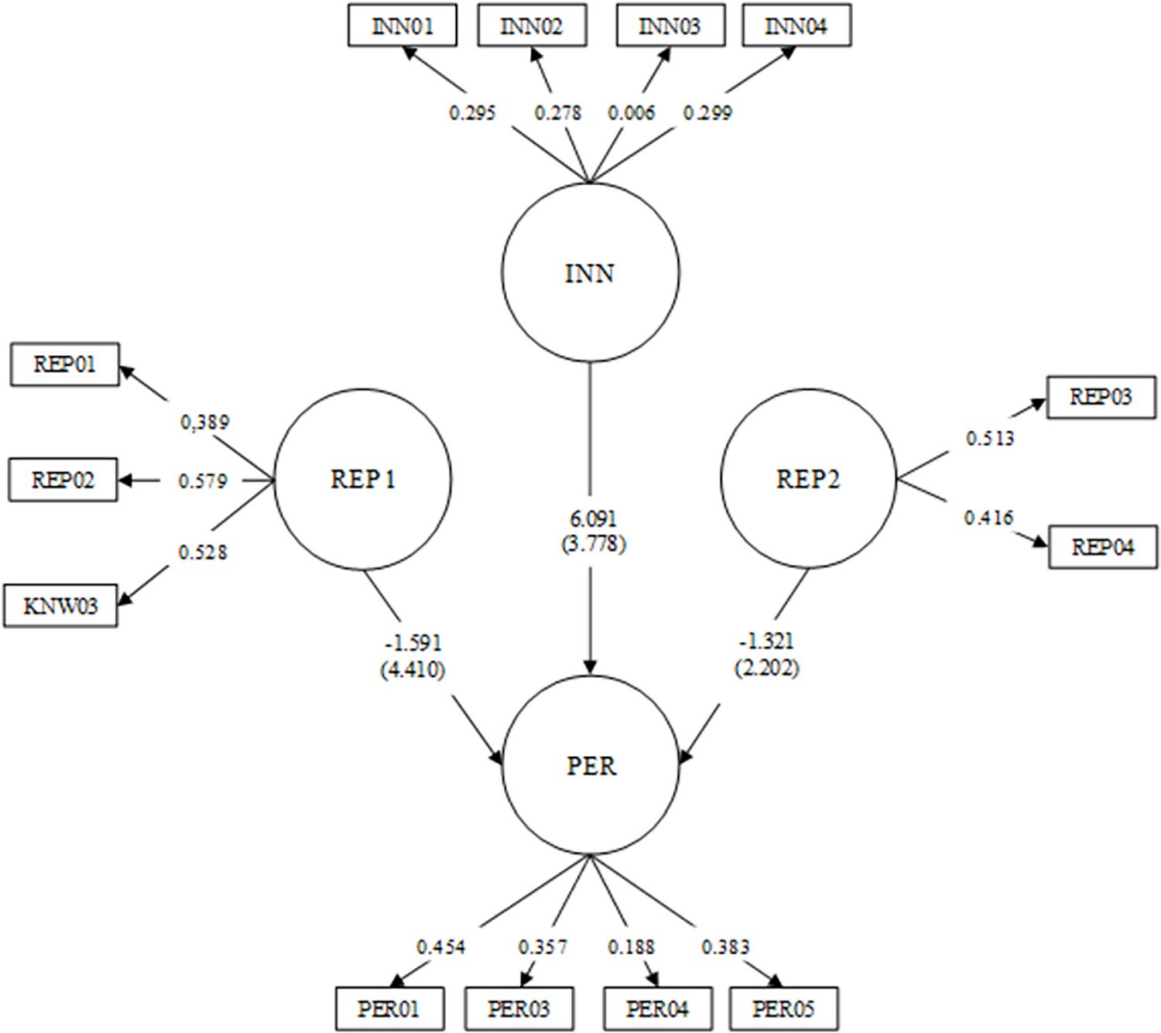
Model of relationship between innovation, Reputation1, Reputation2, and Performance. Standardized loadings of each item are presented between lines going from the factor to the items. Standardized regression coefficients are presented between factors.

A structural model was estimated to test the research hypothesis. Fig 1 shows the factor loadings for each one of the items that compose the latent variables. In addition, the Fig 2 presents the regression coefficients and their standard errors to illustrate the relationship between the latent variables. Just as in the results reported by Hormiga and García-Almeida [3], the model goodness measurements obtained in this second step are lower than those reported in the CFA. However, the absolute criterion (SRMR = 0.058) and parsimony test (0.052) show good results.

### 4.3 Summary of the hypothesis test

Contrary to the findings reported by Hormiga and García-Almeida [3], none of the hypotheses tested in the structural equation model were significant. Table 5 presents the coefficients and t values for each hypothesis.

**Table 5:** Hypothesis validation through the structural equation model.

		Standardized Betas	Z statistic
H1	INN—> PER	4.389	1,612
H2	REP1 —> PER	-1.504	-0,361
H3	REP2 —> PER	-1.569	-0,600

Chi2 (60) = 100,349***; CFI = 0,763; TLI = 0,692 RMSEA(90%CI) = 0.052 (0.033;0,069).

*** p < 0,001; ** p < 0,05; * p < 0,01.

Hormiga and García-Almeida [3] found evidence to confirm the three tested hypotheses with values of significance higher than 99%. In addition, they reported a model with a very good fit (Normed Fit Index (NFI) = 0.887; Tucker-Lewis Index (TLI) = 0.976; RMSEA = 0.037; Goodnes of Fit Index (GFI) = 0.899; Adjusted Goodness of Fit Index (AGFI) = 0.860); however, our estimated model did not find evidence to reject the null hypotheses.

## 5. Discussion

The primary aim of this study was to analyze how MSMEs in the creative sector can build a reputation based on innovation and accumulated knowledge. To this end, Hormiga and García-Almeida´s [3] research in Spain with 130 companies in the service sector was used as a reference point.

The original research tested four hypotheses: 1. The new venture’s reputation is directly and positively associated with organizational performance, 2. The entrepreneur’s stock of knowledge associates directly and positively with the new venture’s reputation. 3. Innovation and knowledge creation associate directly and positively with the new venture’s reputation, and 4. The entrepreneur’s stock of knowledge associates directly and positively with the level of new venture innovation and knowledge creation.

In contrast to the previous research, three hypotheses were tested in this research: 1. Innovation is positively related to reputation, 2. Entrepreneur’s knowledge has a positive impact on reputation, and 3. There is a positive relationship between reputation and corporate performance. These hypotheses were more suitable to the reality of the studied firms. In this sense, this study achieved additional results by refining the initial model with a main contribution here—the consideration of managers’ perspective and realities.

The first main finding of this research is that it was not possible to confirm the original test’s factorial structure. Regarding the factorial analysis, some items were retained because of their theoretical importance for this study. Since this research is still in an exploratory stage, it is suggested to conduct other empirical research to continue adapting and validating the model both for the same sector and for other sectors. In this sense, in the instrument, the authors retained item INN04 because it captures the ability of the company to listen to and manage changes to improve its service. This ability is especially important for entrepreneurs in their initial phase [14] since it is fundamental for the performance of new organizations and demonstrates the flexibility and adaptation of a new company, compared to those already established [104]. Item KNW03 was retained because it evaluates the importance of expertise in the sector [105] since expertise recognition impacts the reputation and overall results of the company [106]. Furthermore, REP04 was retained because it addresses the issue of customer loyalty [107,108] and could even include investors [109]. These adjustments refined the model structure to be applicable for MSMEs. However, further research is suggested in this regard.

The factorial analyses suggested dismissing the technology dimension, which, though not included in the hypotheses, constitutes one of the five original dimensions of the instrument. Moreover, the dimension of entrepreneur’s knowledge did not emerge in the analysis, and the two remaining items were included in the reputation dimension.

It is noteworthy that reputation was divided into two dimensions. Reputation1 relates to the perception that entrepreneurs have of the degree of reputation achieved by the company up to the time it was evaluated [110], considering their years of experience [111], that is, more related to internal management [112]. Since the focus goes from the inside to the outside, it will be understood as a “desired image” by what was found by Walker [10], who states that the image of an organization is described as an internal image projected to the external public. Moreover, Reputation2 relates to customers’ recommendation of the service and their loyalty [113], which is congruent with the external management of customers, that is, this factor retains the name “reputation” in agreement with the studies of O’Connor and Assaker [114] and Sharma and Joshi [115], who relate a reputation to loyalty. In summary, it was not possible to find evidence for the relationship between Innovation, Reputation1, Reputation2, and the Performance of new firms. This information is of great importance for entrepreneurs who develop audiovisual, cultural, publishing, digital media, and advertising activities, among others (related to the orange economy), as they need to adapt to changes in the environment and context and propose actions that allow them to differentiate and innovate in such a competitive environment. While also focusing on reputation management internally and externally, customers prefer to choose organizations that demonstrate experience and knowledge in the value proposition they deliver to their customers.

The second main finding of this research is that despite confirming the factorial structure of the instrument, no evidence was found to support the three proposed hypotheses in this research. Regarding the first hypothesis, innovation is positively related to reputation, the statistical analysis was not conclusive. The concept of innovation in the orange sector requires a broader idea. The MSME managers expressed in the interviews that their success in making better processes, products, and services was based on their talents and not on the technology available. However, if asked about innovation, they did not acknowledge their practices as innovative. They assumed these practices are just part of daily activities and for this reason they did not realize that something new or with more value was happening in their operations. For this reason, in the model, the relationship between innovation and reputation was not significant.

On the other hand, the results of the second hypothesis, entrepreneur’s knowledge has a positive impact on reputation, were not significant either. Once again, managers in these firms do not separate their idea of talent and knowledge. Particularly, when they are talking about how they manage the business, they express the view that this way of doing business was formed during the building of the business, or learned from relatives or previous managerial experiences. They do not connect the acquired knowledge of doing business with the current status of the business. The connecting relationship between entrepreneurs’ knowledge and reputation was not considered by managers as the results demonstrated.

Finally, the third hypothesis, there is a positive relationship between reputation and corporate performance, was not significant. In this case, it was notable that managers cared about the reputation of their business due to its relationship to the way they conduct their business. It means again, they do not distinguish between what is talent or technical knowledge and what is how others see them. In this sense, they do not see whether they are known in the sector because of the way they do business or because the performance of the firm is based on their ability to gain customers. In this regard, if the manager does not pay attention to the reputation of the firm, they are not going to relate it with achieving performance.

This study contributes to knowledge by shedding new light on the conceptualization of reputation building [116] based on the internal knowledge of an organization [117]. It proves that tacit knowledge is not only the ability to innovate. It is relevant to be aware of one’s own abilities and knowledge, even when the performance of the firm is achieved because of those elements. Tacit knowledge, as Ganguly [34] pointed out, generates a competitive advantage for a company [118]. Although reputation is an existing theoretical construct with extensive research and studies [119,120], it was not known how the relationship between technical knowledge and innovation could be energizing for those companies where resources were limited and sources of capital were often inconvenient [121]. Thus, despite the fact that a small company does not have financial muscle, which is one of the most frequently reported difficulties of companies in the creative and cultural sector [122], it can be seen that reputation requires self-acknowledgment by managers.

## 6. Conclusions

To conclude, this study contributes to the existing literature by expanding the theoretical knowledge about small and medium-sized enterprises that do not have a technological base for their operation and that, as in the case of the orange economy, have knowledge and each entrepreneur’s talent as primary pillars [123]. Thus, the theory now acquires a broader dimension by considering reputation as a phenomenon that receives financial resources [124] and skills [125]. This approach greatly increases scalability to enable the interpretation of returns in companies based on creativity, which, as recognized by the Departamento Nacional de Planeación (National Planification departmet) (DNP in Spanish) in its diagnosis contained in CONPES 4090 [122], presents shortcomings in training in the same way that the supply of the educational system presents gaps in terms of quality, relevance, and deficit of the programs offered, especially in rural regions of Colombia.

On the other hand, the model was tested in companies in the orange economy with less than five years of existence and in companies where the experience transmitted in the development of the art, service, or product delivered to the client plays an essential role in generating credibility. Thus, reputation is an extrinsic signal that develops over time and the shared valuations of the company’s users and stakeholders [116,117]. In this sense, to build the company’s reputation takes time; however, in the services sector and products related to the orange economy, the close relationship also requires an awareness of the business scope. It is not enough to develop the mouth-to-mouth strategy to get the customers. Therefore, it is suggested to implement customer-centric strategies and adapt the customer journey, starting with the buyer persona (customer profile or target segment) to define and adapt each strategy and activity of the customer relationship process. It is also essential to consider the self-understanding of managers related to the core competencies of the firm and have a loyalty process that will influence the repurchase and, in the end, directly impact the company’s performance.

In addition to all of the above, it is established that the promotion of sectors such as creative talent requires strengthening the core business’s entrepreneurial, managerial, and strategic management skills, as recognized by the Ministry of Culture [123]. In this sense, and from a governmental point of view, while promoting a sector is necessary, it is also necessary to focus resources that can achieve a more significant potential impact when they reach the capacity building of individuals, who are ultimately their main competitive advantage in their sector, as stated in this study. For policymakers, this finding leads to the conclusion that there is a need to create capacity-building programs as a priority over programs or mechanisms that only serve to promote and disseminate the dynamics of the sector and not strengthen it.

Finally, this research provides additional information within the legal framework of the orange economy in Colombia, where seven strategies for the management of the sector by the government are contemplated in Law 1834 of 2017 [89]. These strategies include information, institutions, industry, infrastructure, integration, inclusion, and inspiration. However, none of them contemplate the individual strengthening mentioned above. Another critical part in the strengthening of this sector has been the CONPES 4090 [122], which proposes strategies that sustainably consolidate the orange economy sector in the different territories through four strategic axes. The first axis focuses on the promotion of the identification and recognition of the cultural and economic value of the different artistic expressions. The second axis focuses on the coordination of both public and private actors and the sector. The third axis focuses on improving the environmental and economic conditions to ensure companies’ sustainability in the orange economy sector. Finally, the fourth axis focuses on promoting networking to increase the exchange of cultural and creative goods and services.

Within these four axes, only the third axis focuses on human capital training and the design of specific training programs for creative, cultural, and innovative industries. However, during this time and when the strategies proposed are being consolidated, many companies will go bankrupt. According to the National Planning Department [122], these strategies should be implemented between 2022 and 2027, and it will be necessary to wait until this period is over to know the impact these strategies have had on the economic and social development of the sector.

### 6.1 Limitations and future research

Although the findings of the study are relevant for entrepreneurs in the sector in question, it is essential to continue with the research to identify what other variables could affect the performance of these companies from the perspective of another group of stakeholders, for example, from the perspective of the customer [61]. Longitudinal studies that show business behavior over time could also be implemented to obtain a complete view of the organization and the strategies that can be implemented at each stage of the process. Similarly, studies are suggested that could test and adapt these models to other sectors of the economy that require intensive knowledge, R&D, and fixed or tangible assets or resources to a greater extent and study their behavior, especially in emerging economies that require them. In addition, it is important to point out that this research was carried out at the beginning of the COVID-19 global health emergency that forced all governments to implement protective measures, such as blockade and containment, to contain the spread of the virus, which, in turn, could have affected the performance of the companies surveyed.

## Supporting information

S1 File (R)
